# Analysis of Performance Losses and Degradation Mechanism in Porous La_2−X_ NiTiO_6−__δ_:YSZ Electrodes

**DOI:** 10.3390/ma14112819

**Published:** 2021-05-25

**Authors:** Juan Carlos Pérez-Flores, Miguel Castro-García, Vidal Crespo-Muñoz, José Fernando Valera-Jiménez, Flaviano García-Alvarado, Jesús Canales-Vázquez

**Affiliations:** 1 3D-ENERMAT, Renewable Energy Research Institute, ETSII-AB, University of Castilla-La Mancha, 02071 Albacete, Spain; miguel.castro@uclm.es (M.C.-G.); vidal.crespo@uclm.es (V.C.-M.); josefernando.valera@uclm.es (J.F.V.-J.); 2 Chemistry and Biochemistry Dpto., Facultad de Farmacia, Universidad San Pablo-CEU, CEU Universities, Boadilla del Monte, 28668 Madrid, Spain; flaga@ceu.es

**Keywords:** SOFC, cathode, degradation, perovskite, long-term, ex-solution

## Abstract

The electrode performance and degradation of 1:1 La_2−x_NiTiO_6−__δ_:YSZ composites (x = 0, 0.2) has been investigated to evaluate their potential use as SOFC cathode materials by combining electrochemical impedance spectroscopy in symmetrical cell configuration under ambient air at 1173 K, XRD, electron microscopy and image processing studies. The polarisation resistance values increase notably, i.e., 0.035 and 0.058 Ωcm^2^ h^−1^ for x = 0 and 0.2 samples, respectively, after 300 h under these demanding conditions. Comparing the XRD patterns of the initial samples and after long-term exposure to high temperature, the perovskite structure is retained, although La_2_Zr_2_O_7_ and NiO appear as secondary phases accompanied by peak broadening, suggesting amorphization or reduction of the crystalline domains. SEM and TEM studies confirm the ex-solution of NiO with time in both phases and also prove these phases are prone to disorder. From these results, degradation in La_2−x_NiTiO_6−__δ_:YSZ electrodes is due to the formation of La_2_Zr_2_O_7_ at the electrode–electrolyte interface and the ex-solution of NiO, which in turn results in the progressive structural amorphization of La_18_NiTiO_6−__δ_ phases. Both secondary phases constitute a non-conductive physical barrier that would hinder the ionic diffusion at the La_2−x_NiTiO_6−__δ_:YSZ interface and oxygen access to surface active area.

## 1. Introduction

Solid oxide fuel cells (SOFCs) are unique electrochemical devices for combined heat and electricity generation in terms of efficiency and environmentally friendly. SOFCs operation requires the development of materials with superior performance at the high working temperature of these devices, typically 1073 K. At these temperatures, SOFCs exhibit fast electrode kinetics, high tolerance to catalyst poisoning and even the possibility using alternative hydrocarbon-based fuels via internal reforming [1]. During SOFC operation the electrical and electrochemical performance of such materials are determined by the electrode polarisation resistance, especially at the cathode side as losses related to the large overpotential of the cathodic oxygen reduction reaction (ORR) can be considered as one the most important contributions [2,3,4].

Among cathode materials, strontium-doped lanthanum manganite (LSM), (La_1−x_Sr_x_)_1−y_MnO_3_ (x = 0.05–0.4, y = 0–0.1) has been extensively studied and is the most used air electrode materials in SOFCs [5,6,7,8,9]. LSM materials are predominantly electronic conductors, as Sr^2+^ doping/substitution at the A-site of the perovskite, La^3+^, creates a positive charge deficit that is compensated by manganese oxidation (Mn^4+^) rather than oxygen vacancies creation. LSM exhibits electronic conductivity values as high as 200–300 S cm^−1^ at 1173 K [10,11] but very low ionic conductivity (≈10^−7^ S cm^−1^) [12]. This limitation is covered by the addition of good ionic conductors and, thus, typical cathode compositions include different LSM:YSZ composite mixtures (YSZ, (ZrO_2_)_1–x_(Y_2_O_3_)_x_, with 0.08 ≤ x ≤ 0.1) [5,8,13,14,15,16]. The thermal and chemical compatibility that both materials show at high temperatures makes them the most suitable cathode materials for SOFCs. 

Degradation on SOFCs may be due to a number of parameters that can be classified into intrinsic (materials phase change, product segregation, microstructural changes, fractures, and delamination) [17,18,19,20] and extrinsic (poisoning and/or reaction with other cell materials, humidity, impurities and components in the fuel and/or oxidising agent) [21,22,23,24,25,26,27] In LSM-based SOFCs, one of the most important degradation mechanisms at the cathode relates to chemical reaction between LSM and YSZ at high temperatures to produce insulating lanthanum and strontium zirconates [27,28,29]. Zirconate secondary phases are obtained as reaction product at the electrode-electrolyte interphase and therefore, the cathodic reaction may be hampered by the participation of zirconates in cathodic reaction via an alternative oxygen diffusion process [30,31]. Moreover, this degradation mechanism has a direct detrimental effect on the stability and durability of the triple-phase boundary region (TPB) at the cathode side which leads to performance depletion of the fuel cell [32,33,34,35].

Over the last couple of decades, many efforts have been devoted to the search for alternative electrode materials for SOFC operation, in many cases offering outstanding performances. However, most of the time such novel materials do not transcend as they end up degrading under working conditions; for stationary SOFCs, total degradation objectives must comply power density losses below 25% after 80,000 h which implies a degradation rate of 0.3%/1000 h [36,37]. Durability or degradation studies that evaluate cell performance in the mid- and long-term should be considered in a routine basis when searching for alternative materials despite they are time-consuming and require of repetitive tests. Indeed, long-term durability issues are considered the major challenges that SOFC technology must face to be considered as a real energy production alternative [38,39]. These costly requirements can be partly achieved overusing alternative accelerated lifetime tests (ALTs) to force the operative degradation mechanisms and accelerate the components stress; thus, it would be possible to obtain illustrative and comparably features about the performance losses in the long-term operative SOFC procedure [40,41,42].

Within this context, this research outlines a new degradation study on alternative cathode materials, i.e., La_2−x_NiTiO_6−δ_ [43,44,45], as they exhibited good thermal and chemical stability and a very promising polarisation resistance at 1073 K, ~0.5 Ω cm^2^, which makes them prospective cathode materials. To date, the focus has been put on LSM-based cathodes, but very few previous studies have considered the long-term degradation of alternative cathodes materials [46,47,48,49]. Therefore, as in a previous study, we proposed La_2−x_NiTiO_6−δ_-based cathodes as a potential electrode material according to their promising electrical characteristics, now an accelerated degradation test has been developed to explore their real potential. This test, performed at temperatures higher than the usual working SOFC temperatures, i.e., 1173 K, allow us to shed light on the degradation mechanism involved in the cathodic reactions related with these compounds and define their potential working conditions. 

## 2. Experimental Section

### 2.1. Sample Preparation

La_2−x_NiTiO_6−__δ_ compounds (x = 0 and 0.2) were synthesised by a modified Pechini method [43,44,45]. Compositions were prepared by adding stoichiometric amounts of Ni(NO_3_)_2_·6H_2_O (Aldrich, ≥97%, Madrid, Spain), La_2_O_3_ (Aldrich, 99.99%) and TiO_2_ (Aldrich, ≥99%) to 100 mL of a 1:2 H_2_O: HNO_3_ (Alfa Aesar, 68–70%, Madrid, Spain) solution kept under continuous magnetic stirring. Next, citric acid (99%) was added in a 3:1 citric acid:metal ions molar ratio. After homogenisation, 5 mL of ethylene glycol (Aldrich, ≥99%%) were added to promote polymerisation. The so-obtained resin was cooled down to room temperature, homogenised by powder grinding and heated up to 1073 K for 2 h to remove the organic matter. Finally, the resulting powder was further ground and fired at 1773 K at ≈2 K min^−1^ for 24 h. 

YSZ electrolytes (ca. 1 mm thickness) were fabricated by sintering uniaxially pressed 8%-YSZ (PI-KEM, >99%) discs at 1773 K for 6 h, to produce relative densities higher than 99%. Symmetrical cells were fabricated by coating an active circular area of 6 mm diameter on both sides of the YSZ discs with 1:1 La_2−x_NiTiO_6−__δ_:YSZ slurries based on Decoflux (Zschimmer and Schwarz, WB41, Vila-Real, Spain) as both binder and suspension media. The resulting assemblies were fired at 1423 K for 10 h and subsequently coated with Pt-paste (Fuel Cell Materials) as current collector that was annealed at 1173 K for 1 h, as previously described [44].

The chemical compatibility of the electrode materials, i.e., as synthesized La_2−x_NiTiO_6−__δ_ and YSZ (Pi-KEM), was evaluated using the same 1:1 La_2−x_NiTiO_6−__δ_:YSZ mixture. Then, powders were pelletised and heated in the 1073–1473 K temperature range for 15 h and ambient air.

### 2.2. Characterisation Techniques

Phase purity of the samples was assessed by powder X-ray diffraction (PXRD) analysis using a diffractometer PANalytical’s model X’Pert PRO MRD (Malvern, Worcestershire, UK) with monochromatic Cu Kα1 radiation (λ = 1.5406 Å) operating at 45 kV and 40 mA in the 2θ = 10–80°. Profile fitting of diffraction patterns was performed by FullProf software (2020 version, freeware) [50].

Scanning electron microscopy (SEM) was used to evaluate the macro and microstructure of the samples using a JEOL model JSM-6490LV microscope (Tokyo, Japan) operated at 20–30 kV and equipped with an EDS detector (Oxford Link, Oxford, UK) analyser for energy dispersive spectroscopy (EDS).

Transmission electron microscopy (TEM) studies were carried out using a JEOL model 2100 microscope (Tokyo, Japan) operating at 200 kV and equipped with an Orius Gatan 2 × 2 MPi digital camera (Pleasanton, CA, USA). Specimens were prepared by finely grinding the powders in acetone and further dispersion using an ultrasonic bath. A few drops of the resulting suspension were deposited in a carbon-coated holey Cu-grid (SPI, 200 mesh) and placed on a double-tilt ±25° sample holder. 

Impedance spectroscopy measurements were performed using a Zahner model IM6 system (Gundelsdorf, Germany) in ambient air in the 473–1173 K temperature range, setting 2 h as stabilization time at each temperature stage, using a two-electrode configuration. Polarisation analysis were performed using the previously described symmetrical cells and the same cell configuration at 1173 K in air for 300 h. All impedance measurements were conducted under potentiostatic conditions at an amplitude voltage of 50 mV in the 10^5^–1 Hz frequency range. Quality of the impedance data obtained was evaluated using Kramers–Kronig test (KKtest freeware 1.01 version [51,52]). Impedance spectra were fitted using ZView 2.0 software (Scribner Assoc., Southern Pines, NC, USA).

Post-mortem geometrical analysis of La_2−x_NiTiO_6−__δ_:YSZ electrodes was carried out by image processing with Fiji software [53]. SEM micrographs were obtained along the cross-section of each symmetrical cell after the long-term experiments. Three images for each compound were analysed considering an electrode surface area per image approximately of 720 μm^2^ (≈60 μm × 12 μm). A rectangular electrode area was cropped from the original image to avoid contributions due to the electrolyte, the electrode surface, etc. The Otsu thresholding technique [54] was used to the electrode surface segmentation into two classes and minimising the variance inter-class: foreground (solid in white colour) and background (pore in black colour). The resulting parameters were A_t_ (total area from the cropped rectangle), A_p_ (whole porous area), ε (porosity ε = A_p_/A_t_), the average size of pore surface (A_s_), S_v_ is the sphere volume which is obtained from A_s_ and the fractal dimension (D).

## 3. Results and Discussion

### 3.1. Structural Characterisation

**Figure 1:**
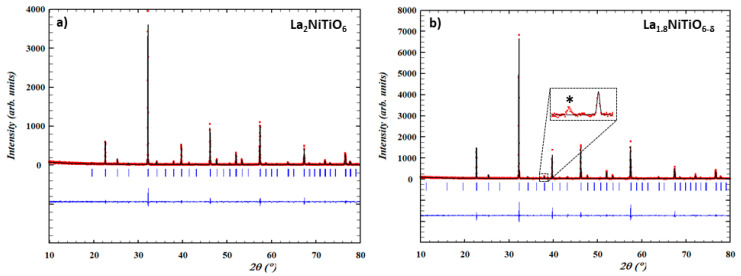
Experimental (red circles), calculated (black continuous line), their difference (blue line) and Bragg peaks position (blue vertical bars) for XRD patterns of as prepared (**a**) La_2_NiTiO_6_ and (**b**) La_1.8_NiTiO_6−δ_ at 1773 K for 24 h in air.

Figure 1 shows the XRD patterns of as synthesized La_2−x_NiTiO_6−__δ_ (x = 0, 0.2) compounds. Both were obtained as single phases and were indexed using LeBail method as double perovskite structures with P2_1_/n (#14) and P2_1_ (#4) monoclinic symmetry groups for x = 0 and x = 0.2, respectively. Lattice parameters are a = 5.5808(2) Å, b = 5.5910(8) Å, c = 7.8875(3) Å and β = 90.04775° for La_2_NiTiO_6−__δ_ (V = 246.113(11) Å^3^) and a = 5.5767(3) Å, b = 5.5785(3) Å, c = 7.8779(5) Å and β = 90.0562(39)° for La_1.8_NiTiO_6−__δ_ (V = 245.078(23) Å^3^). Therefore, vacancy creation at the A-site of the primitive perovskite does not significantly change the symmetry characteristics, except for a slight volume reduction consistent with a mixed charge compensation mechanism of oxygen vacancies creation ($V_{O}^{∙∙}$, Equation (1)) and nickel oxidation (Ni^3+^, Equation (2)), being the former the predominant as previously described [43,44]:

(1) $${\mathrm{La}}_{\mathrm{La}}^{x}+\frac{3}{2}{\;O}_{o}^{x}\to {\;V}_{\mathrm{La}}^{\prime \prime \prime }+\frac{3}{2}{\;V}_{O}^{∙∙}+\frac{1}{2}{\;\mathrm{La}}_{2}O_{3}$$

(2) $$2{\mathrm{La}}_{\mathrm{La}}^{x}+6{\mathrm{Ni}}_{\mathrm{Ni}}^{x}+\frac{3}{2}{\;O}_{2}\to 2V_{\mathrm{La}}^{\prime \prime \prime }+6{\mathrm{Ni}}_{\mathrm{Ni}}^{∙∙}+{\;\mathrm{La}}_{2}O_{3}$$

In the case of x = 0.2 composition a very weak peak at 2θ = 37.2° indicates that NiO is present in very small amount, being assigned to its (111) reflection; no additional peaks corresponding to this phase can be identified in the pattern background. As previously pointed out [43,44], secondary NiO phase could come from a small fraction of unreacted product or, more likely, from the structure ex-solution. Although no diffraction peaks of Ni metal are found, it cannot be totally discarded that the mechanism involves Ni metal ex-solution from the perovskite structure [55,56,57] and further oxidation to NiO, rather than direct NiO ex-solution [58], as described in other perovskite-related compounds. This is a consequence of the charge compensation mechanism due to the cation vacancies creation in the A-site, alternatively to the formation of larger number of oxygen vacancies and the concentration increase of trivalent nickel content from oxidation in the structure.

**Figure 2:**
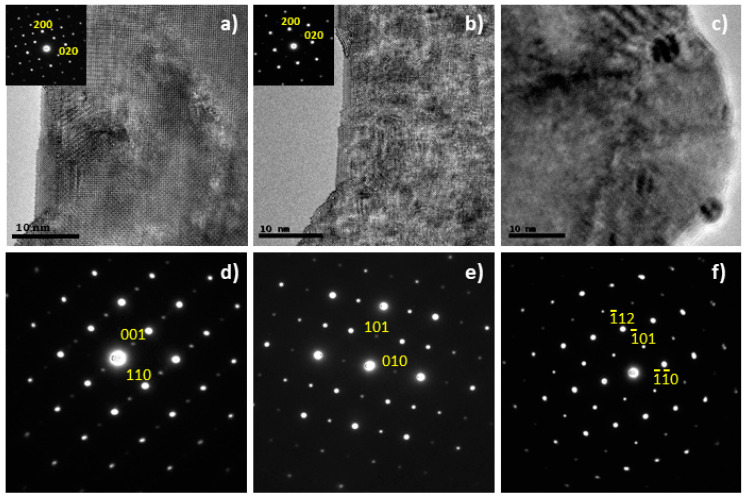
HRTEM images showing a view down the [001] in La_2_NiTiO_6−__δ_, (**a**) time = 0 and (**b**) time = 60 s. Under beam irradiation, the perovskite undergoes ion rearrangement resulting in a more disordered matrix. The insets correspond to the SAED patterns that also highlight such change. (**c**) TEM image of as prepared La_1.8_NiTiO_6−__δ_ revealing the presence of ex-soluted NiO nanoparticles. SAED patterns showing views down the (**d**) $\left[1\bar{1}0\right]$, (**e**) $\left[10\bar{1}\right]$ and (**f**) $\left[\bar{1}1\bar{1}\right]$ zone axes of La_2−x_NiTiO_6−__δ_ phases.

The selected area electron diffraction (SAED) patterns can be indexed according to double perovskites (Figure 2) for both La_2−x_NiTiO_6−__δ_ phases, in good agreement with the XRD results. However, the TEM images reveal the presence of ex-soluted NiO nanoparticles (5 nm) in the case of the x = 0.2 composition, which explains the weak reflection at 2θ = 37.2° observed by XRD (Figure 2c). For the stoichiometric composition, no ex-soluted nanoparticles were observed although the structure proved to be rather sensitive to the mild reducing environment inside the TEM column (200 kV irradiation under high vacuum conditions) and structural changes were observed in situ after a few minutes of beam exposure (Figure 2). Despite the reflections corresponding to the primitive perovskite remained in the SAED patterns, the HRTEM images revealed a certain degree of ion rearrangement that resulted in a less crystalline structure. This could be interpreted as rather sensitive structures which may be prone to degradation in the working conditions of a SOFC as discussed below.

**Figure 3:**
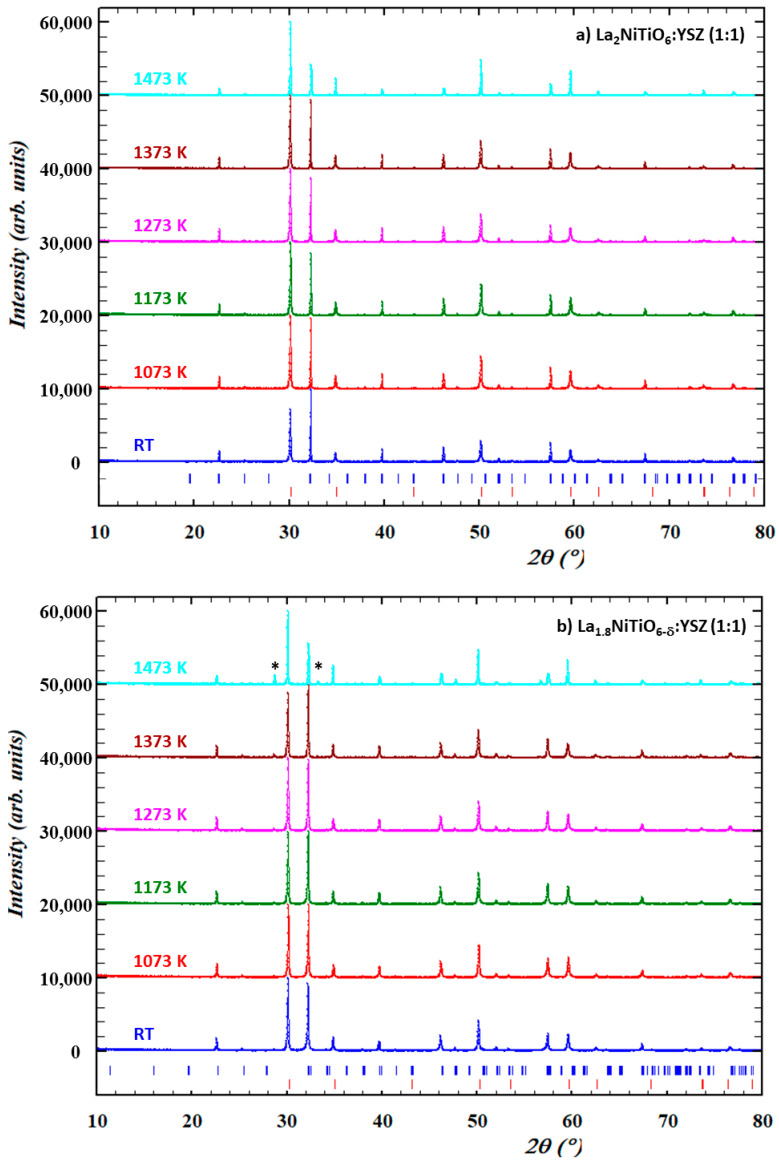
XRD patterns of 1:1 mixture of La_2−x_NiTiO_6−δ_ (**a**) x = 0 and (**b**) 0.2 with YSZ in air at RT (blue), 1073 K (red), 1173 K (green), 1273 K (pink), 1373 K (brown), and 1473 K (cyan). Bragg peaks (vertical bars) of corresponding phases are found at the bottom (La_2−x_NiTiO_6−δ_ in blue and 8-YSZ in red).

Prior to their electrochemical performance, the chemical compatibility of both materials with YSZ electrolyte was assessed under air atmosphere in the 1073–1473 K temperature range to cover both SOFC working and processing temperatures and even higher temperatures to explore the stability limits of the mixture. XRD patterns of these samples (Figure 3) do not reveal any significant structural changes, decomposition, or reaction between the electrode components up to 1373 K when compared with the starting material at room temperature (RT). Therefore, this could be interpreted as these materials being compatible with the working conditions in a typical SOFC.

On the other hand, at temperatures as high as 1473 K, both materials exhibit significant structural changes, as suggested by peak broadening and shift, as well as changes in the relative intensity (Figure S1). Particularly, La_1.8_NiTiO_6−__δ_:YSZ composition shows additional diffraction peaks at ≈28.6° and 33.4° assigned to the La_2_Zr_2_O_7_ compound [59,60]. Therefore, the reaction of La_1.8_NiTiO_6−__δ_ with YSZ limits the usefulness [61] of the composite cathode and interface to temperatures below 1473 K, as zirconates exhibit low conductivity and are detrimental for SOFC operation. Moreover, it should be noted that as these solid-state reactions are diffusive processes dictated by Fick’s Law, more prolonged interaction time may have an equivalent effect at lower temperatures and thus, this must be considered for potential applications of this composite material. 

### 3.2. Electrical and Electrochemical Performance

According to previous results [44], La_2−x_NiTiO_6−__δ_ (x = 0, 0.2) compounds exhibit quite similar behaviour under cathodic conditions, with modest conductivity values ≈1.6 and 1.9 mS cm^−1^ at 1073 K in air for La_2_NiTiO_6−__δ_ and La_1.8_NiTiO_6−__δ_, respectively, and very promising polarisation resistance of ≈0.5 Ω cm^2^ at 1073 K in oxygen for La_1.8_NiTiO_6−__δ_. This polarisation resistance is even better than the state-of-the-art La_1−x_Sr_x_MnO_3_-based cathode materials [13,31,62,63]. Therefore, it is convenient to explore deeply the potential use of this materials under more demanding conditions in the long-term. 

Polarisation impedance experiments were performed in both La_2−x_NiTiO_6−__δ_:YSZ/YSZ/La_2−x_NiTiO_6−__δ_:YSZ symmetrical cells (x = 0, 0.2). The resulting spectra were analysed to assure their quality prior to the complex nonlinear square (CNLS) fitting procedure. Mainly, poor data would be obtained from non-stationary systems, errors coming from the measurement system and too large perturbations applied. Data fulfilment with Kramers–Kronig (KK) relations guarantees the good quality of the impedance data [64,65,66]. Figures S2–S5 shows the results obtained from the KK test for La_2_NiTiO_6_ and La_1.8_NiTiO_6−__δ_, respectively. In general, points distribute randomly around the frequency axis (Figures S2 and S4) in the relative difference plots (ΔZ_i_) although a trend can be observed at around 2 KHz for La_2_NiTiO_6_ when increasing the temperature, which is, in any case, less than 0.5% or below of residuals. Therefore, the impedance data are considered KK transformable. The KK transform data in the complex plane can also be observed for La_2_NiTiO_6_ and La_1.8_NiTiO_6−__δ_ (Figures S3 and S5); these plots highlight the good agreement of the KK transformation and experimental data, with χ^2^ values within 9.5 × 10^−8^ to 9.4 × 10^−7^ in the 1173–973 K temperature range (2.5 × 10^−6^ at 923 K) and therefore, confirm the data quality.

**Figure 4:**
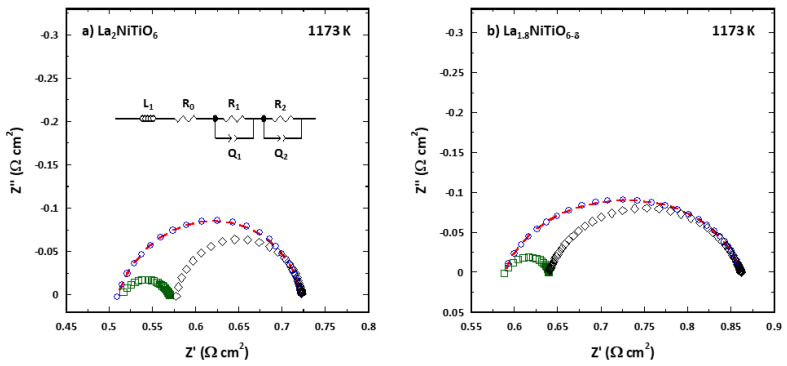
Impedance spectra of La_2−x_NiTiO_6−__δ_:YSZ/YSZ/La_2−x_NiTiO_6−__δ_:YSZ symmetrical cells for (**a**) x = 0 and (**b**) 0.2, at 1173 K and air atmosphere, fitted to equivalent circuit shown in the inset. Experimental data and fitted spectra are depicted as blue circles and red dotted line, while deconvolution of R_1_Q_1_ and R_2_Q_2_ elements appears as green squares and black diamonds.

To assess the polarization performance of La_2−x_NiTiO_6−__δ_:YSZ/YSZ/La_2−x_NiTiO_6−__δ_:YSZ the impedance data of the corresponding symmetrical cells were analysed using the CNLS fitting routine of ZView 2.0 to obtain the corresponding equivalent circuits. An inductance element, L, was used to simulate the wiring of the experimental setup. The 8-YSZ electrolyte was modelled as a series ohmic resistance, R_O_. Asymmetric depressed semicircle observed in impedance plot includes several processes in the polarization resistance of the cells. This arc can be satisfactorily described using two in series RQ elements (resistance, R, and constant phase element, CPE, in parallel combination). Using Boukamp’s equivalent circuit notation, it can be represented as LR_O_(R_1_Q_1_)(R_2_Q_2_) (see inset in Figure 4). Constant phase element, with a pseudocapacitance Q, was employed instead of simple capacitor, C, to account for the distributed relaxation frequency of the ceramic electrode, related to dispersion of the f_max_ values of the corresponding electrochemical processes. The true capacitance value associated to this element is obtained by C = R^(1−n)/n^Q^1/n^, being R the resistance and n-coefficient the deviation degree respect to a pure capacitor for which n = 1.

Figure 4 shows a typical Nyquist impedance diagrams of both La_2−x_NiTiO_6−__δ_:YSZ/YSZ/La_2-x_NiTiO_6-__δ_:YSZ symmetrical cells at 1173 K. Impedance spectra were fitted to the equivalent circuit mentioned above (depicted in inset), with their corresponding fitting (red dotted line). The magnitude of the R_1_Q_1_ and R_2_Q_2_ elements is represented in the corresponding plot, as green and black semi-arcs, respectively. Considering both the real and the imaginary component of |Z|, the error between experimental and fitting was less than 1% in the whole temperature range for both compositions and therefore, fitting of impedance data is considered of good quality. 

**Figure 5:**
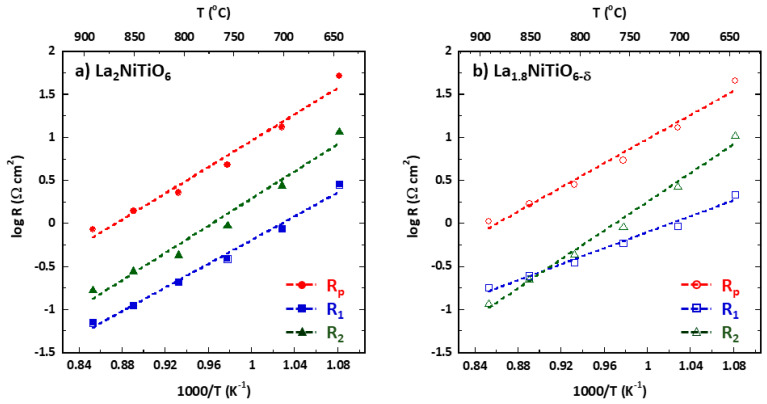
Arrhenius plot and corresponding fittings (dashed line) of La_2−x_NiTiO_6−__δ_:YSZ/YSZ/La_2−x_NiTiO_6−__δ_:YSZ symmetrical cells for (**a**) x = 0 (solid symbols) and (**b**) 0.2 (empty symbols) in the 1173 to 923 K temperature range. Rp (red circles), circuit resistance #1 (R_1_, blue squares), and #2 (R_2_, green triangles).

Figure 5 shows the Arrhenius plot for R_p_, R_1_, and R_2_, obtained in the 1173 to 923 K temperature range, obtained by fitting the corresponding impedance data to the previous equivalent circuit. Table 1 summarises the values obtained for the activation energy, E_act_, and medium capacitance. As the temperature decreases, the polarisation resistance, R_p_ = R_1_ + R_2_, increases as occurs for both R_1_ and R_2_, with the latter being the main contribution to the Rp increase for La_2_NiTiO_6_/YSZ in the entire temperature range; in the case of La_1.8_NiTiO_6−__δ_/YSZ, R_1_ contribution is dominant in the high temperature range.

The high frequency arc (R_1_Q_1_ element) exhibits a medium capacitance of 8.70 × 10^−^^5^ F cm^−^^2^ and 5.67 × 10^−^^5^ F cm^−^^2^ for La_2−x_NiTiO_6−__δ_ (x = 0, 0.2), respectively. Considering the high capacitance value, a contribution coming from the YSZ grain boundary must be discarded because of a lower capacitance is expected accordingly to previous reports (<10^−^^8^ F cm^−^^2^) [14,15,16]. Therefore, this contribution is assigned to the ionic transport of oxygen and/or oxo-compounds across the interface of electroactive material and the electrolyte, i.e., La_2−x_NiTiO_6−__δ_/YSZ, including both the YSZ component from the electrode and the electrolyte [7,31]. Although it is difficult to unambiguously separate both processes, the activation energy (E_act_) can provide information regarding this. The E_act_ values of R_O_ and R_1_ in La_1.8_NiTiO_6−__δ_/YSZ/La_1.8_NiTiO_6−__δ_:YSZ, are 0.9 and 0.92 eV, respectively, likely indicating that the process related with R_1_Q_1_ element of this electrode would be mainly the ionic transport in the YSZ electrolyte matrix. Moreover, the processes involved in the R_1_Q_1_ semi-arc are significantly rate limiting for La_2_NiTiO_6_/YSZ electrode due to its higher E_act_, 1.37 vs. 0.92 eV. On the other hand, and according to the Arrhenius plot, these processes show significantly lower resistance for La_2_NiTiO_6_/YSZ electrode than for La_1.8_NiTiO_6−__δ_/YSZ one at high temperature (1173–1073 K).

The low frequency arc (R_2_Q_2_ element) exhibits a medium capacitance of 1.02 × 10^−^^4^ and 2.82 × 10^−^^4^ F cm^−^^2^ for La_2−x_NiTiO_6−__δ_ (x = 0, 0.2), respectively. Considering the corresponding E_act_, 1.57 and 1.66 eV, and the significant ionic conductivity of La_2−x_NiTiO_6−__δ_ compounds [43,44], this arc can be ascribed to a combination of dissociative adsorption of oxygen and bulk and surface diffusion of oxygen species [15,21,67]. According to the E_act_, 1.57 vs. 1.66 eV, both processes show quite similar rate limitations for both composite electrodes. Moreover, they are less impeded for La_1.8_NiTiO_6−__δ_/YSZ electrode in the highest temperature range.

### 3.3. Long-Term Performance

**Figure 6:**
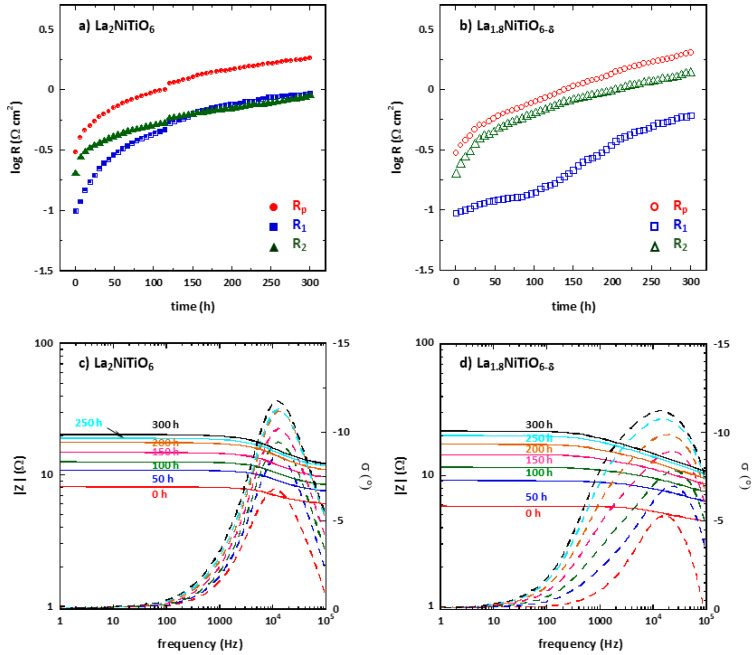
Time degradation of R_p_ (red), R_1_ (blue) and R_2_ (green) of La_2−x_NiTiO_6−__δ_:YSZ/YSZ/La_2−x_NiTiO_6−__δ_:YSZ symmetrical cells for (**a**) x = 0 and (**b**) x = 0.2 at 1173 K and ambient air. Element circuit #1 (R_1_, blue) and #2 (R_2_, in green). Bode plots for (**c**) x = 0 and (**d**) 0.2 at 1173 K and ambient air at different times.

Figure 6a,b shows the polarisation resistance Rp as a function of time for La_2−x_NiTiO_6-__δ_:YSZ/YSZ/La_2−x_NiTiO_6−__δ_:YSZ symmetrical cells (x = 0, 0.2) in ambient air at 1173 K for 300 h. Fitting data was developed using the same equivalent circuit above explained, LR_O_(R_1_Q_1_)(R_2_Q_2_) (Figure 4 inset). Although the initial values of R_1_ and R_2_ were slightly different to those of the cells tested for Arrhenius plots, which may be due to differences in the electrode morphology. 

Initially, the main contribution to the cell resistance is due to R_2_Q_2_ component in both compositions. However, the contribution of R_1_Q_1_ and R_2_Q_2_ elements to the Rp magnitude equals after ≈145 h. The cell performance decreases with time at a rate of ≈0.035 and 0.058 Ωcm^2^ h^−1^ for x = 0 and 0.2 samples, respectively. No similar long-term degradation test was found in the bibliography, but for a symmetrical cell with YSZ electrolyte and LSM:YSZ electrodes, which shows a degradation rate of 3.04 mΩcm^2^ h^−1^ at 923 K. The degradation of La_2−x_NiTiO_6−__δ_:YSZ-based electrodes is higher, although the testing conditions in the present work are significantly more demanding. The most significant magnitude change is found for R_1_ resistance of La_2−x_NiTiO_6−__δ_:YSZ electrode but not very different from the other values. From the Bode plot (Figure 6c,d), it is possible to determine which element of the fitted circuit contributes more to the cell degradation. For these symmetric cells with La_2_NiTiO_6−__δ_:YSZ composite electrode, a progressive degradation of both elements’ circuit, R_1_Q_1_ and R_2_Q_2_, is observed as the resistance increases, mainly in the processes occurring at ≈1.2 kHz which is related with the R_2_Q_2_ element. On the other hand, La_1.8_NiTiO_6−__δ_:YSZ based symmetrical cell changes are more significant, showing a shift of the arc corresponding to R_2_Q_2_ element towards lower frequencies, while both elements increase their resistance similarly. 

To monitor whether any significant changes occurred under the SOFC working conditions tested that could explain the relatively large degradation observed in these materials, La_2−x_NiTiO_6−__δ_ (x = 0 and 0.2) starting materials and 1:1 La_2−x_NiTiO_6−__δ_:YSZ composite mixtures were heated up to 1173 K for 250 h. Using the as synthesised materials as reference, it is possible to find some changes after 250 h for La_2−x_NiTiO_6−__δ_ compositions by XRD; there is a marked peak shift, which is accompanied by peak broadening. This is consistent with the amorphization/crystallinity loss under the reducing mild conditions of TEM beam exposure. This effect is especially pronounced for La_1.8_NiTiO_6−__δ_ (Figure S6). Besides, it appears a NiO secondary phase (2θ = 37.2°) related to ex-solution processes in La_1.8_NiTiO_6−__δ_ as described later, appears too. Despite this, the main structural characteristics of the initial phases remain after the heating process.

Regarding 1:1 La_2−x_NiTiO_6−__δ_:YSZ composite electrode (Figure S7), the most significant change observed in both compositions is the appearance of two additional peaks at 2θ = 28.7° and 33.4° after 250 h at 1173 K that, in good agreement with the previous discussion regarding the formation of zirconate at the electrode–electrolyte interface, correspond to the (222) and (400) diffraction peaks of La_2_Zr_2_O_7_ compound. As previously discussed, the compatibility tests gave no indication of reaction between La_2−x_NiTiO_6−__δ_ and YSZ below 1473 K, which now appears as a clear evidence of the electrode degradation and/or components reaction. The zirconate phase formation could be related to the R_1_Q_1_ element degradation via formation of an insulating barrier compound hindering the physical processes involved at the electrode-electrolyte interface. More precisely, this secondary phase would hamper the ionic transport processes due to its poor ionic conductivity compared to YSZ compound [68,69,70], increasing the R_p_ and, consequently, decreasing the cell performance. Additionally, diffraction peaks of La_2−x_NiTiO_6−__δ_ compounds appears significantly broader and shifted towards higher angles (Figure S4), pointing out to an amorphization/crystallinity loss or even progressive degradation of initial phases in line with degradation results of pure phases.

The presence of impurities in air can be considered as the main reason for the general degradation observed for R_2_Q_2_ elements, related to the adsorption of oxygen and diffusion of oxygen species in both La_2−x_NiTiO_6−__δ_:YSZ composite electrodes. This phenomenon appears to be more significant for La_1.8_NiTiO_6−__δ_:YSZ electrode. The source of this problem could be the segregation and/or formation of oxides such as NiO and La_2_Zr_2_O_7_ with might reduce the active surface by blocking the triple phase boundary. This situation would impede the gas access to the active sites and therefore, increase the contribution of R_2_Q_2_ elements to the polarization resistance. As this circumstance is especially aggravated for La_1.8_NiTiO_6−__δ_:YSZ material, it would appear as one of the main reasons for accelerated cell performance degradation.

**Figure 7:**
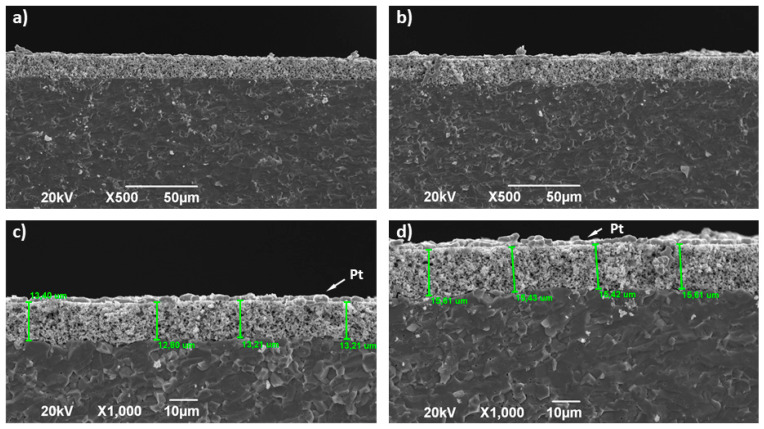
SEM micrographs of La_2−x_NiTiO_6−__δ_:YSZ composite electrodes and interface with YSZ electrolyte (**a**,**b**) ×500 and (**c**,**d**) ×1000 for La_2_NiTiO_6−__δ_ and La_1.8_NiTiO_6−__δ_, respectively.

After the polarisation experiments finished, the cross-section of La_2−x_NiTiO_6−__δ_:YSZ electrodes with YSZ electrolyte was investigated by SEM (Figure 7) searching for additional degradation factors. Typical micrographs show quite good adhesion between the composite electrode and the electrolyte, without significant voids or cracks in the interface. Besides, the electrode thickness (≈13.2 and 15.5 μm for La_2_NiTiO_6_ and La_1.8_NiTiO_6−__δ_, respectively) and porosity distribution exhibit quite homogenous distribution by visual inspection of the micrographs. Therefore, and according to conventional characterisation of SOFC electrodes, it seems that no significant performance loss can be ascribed to the degradation of the physical characteristics and morphology of the electrodes.

**Figure 8:**
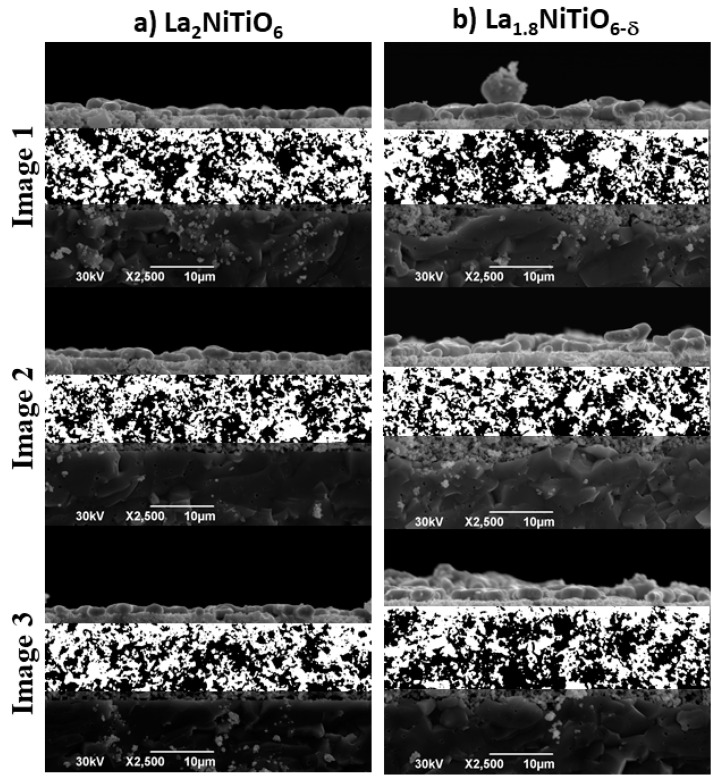
SEM micrographs of La_2−x_NiTiO_6−__δ_:YSZ composite electrodes for (**a**) La_2_NiTiO_6−__δ_ and (**b**) La_1.8_NiTiO_6−__δ_ and segmentation by Otsu thresholding (white and black for foreground and background, respectively).

The morphology and geometry of the pores were characterised by image processing of post_−_mortem SEM cross sections of electrodes (Figure 8 and Table 2). The surface morphology of the pores, which can be described by its fractal dimension (D), provide information about gases accessibility to the electrode surface area and the reaction kinetics and therefore, about the potential cell performance and the cell polarisation [71]. The fractal geometry description of the pore surface in both La_2−x_NiTiO_6−__δ_-based electrodes is almost the same, with a less than 1% difference and a fractal dimension correlation (r^2^) above 0.99 for nine boxes. Therefore, the fractal results of pore morphology suggests that both electrodes are morphologically alike [72], which dismisses any performance difference between both materials in terms of porosity (ε) morphology. 

On the other hand, a Euclidean geometry pore analysis shows up to 24% and 34% higher mean values, $\bar{x}$, of pore area (A_s_) and sphere volume (S_v_) for La_1.8_NiTiO_6−δ_:YSZ electrode, respectively. However, the porosity difference between both electrodes is lower than 6% (49 vs. 52%), with porosity values in range to previous works [73,74].

Although the La_1.8_NiTiO_6−δ_:YSZ electrode has less porosity, the corresponding sphere volume is significantly higher (1.146 vs. 0.753 μm^3^). Larger pore volume means lower surface active area and therefore the dissociative oxygen process of the La_1.8_NiTiO_6−δ_:YSZ electrode should be lower than the same for La_2_NiTiO_6_:YSZ, as observed in the final stage of the degradation test (Figure 6), when the resistance due to R_2_Q_2_ element is higher.

**Figure 9:**
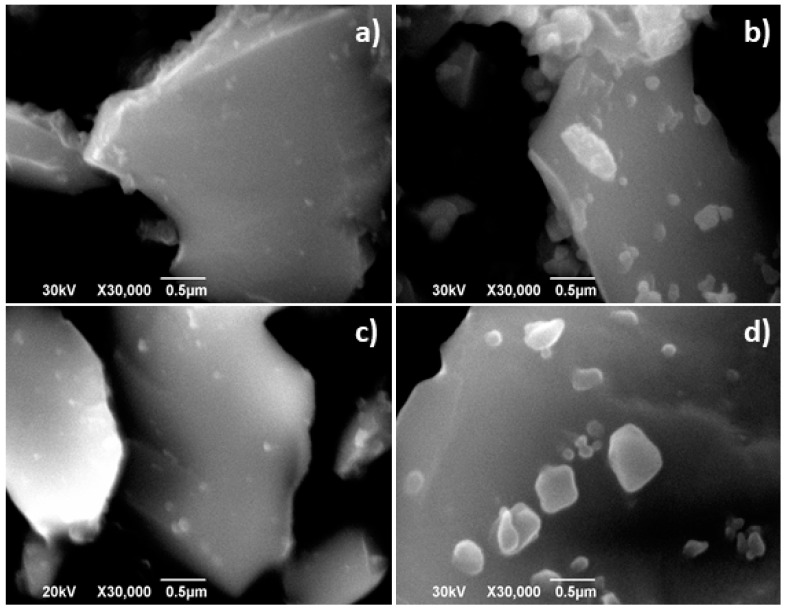
SEM images corresponding to (**a**) as prepared and (**b**) La_2_NiTiO_6_ after annealing at 1173 K for 300 h and (**c**) as prepared La_1.8_NiTiO_6−__δ_ and (**d**) after annealing at 1173 K for 250 h, respectively.

The SEM and TEM characterisation performed in La_2−x_NiTiO_6−δ_ powders annealed in air at 1173 K for 250 h reveals marked changes compared to the initial structure and microstructure. The SEM images reveal that ex-soluted nanoparticles appear in the surface of La_2_NiTiO_6_, whilst initially the crystal surface barely showed the presence of such particles (Figure 9a,b). In the case of La_1.8_NiTiO_6−__δ_, initially the ex-soluted nanoparticles are clearly visible and their number significantly increases upon time and moreover, the average size is larger (Figure 9c,d). 

**Figure 10:**
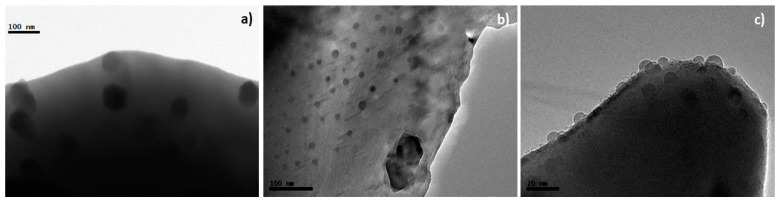
TEM images obtained after annealing at 1173 K for 250 h for (**a**) La_2_NiTiO_6_ and (**b**,**c**) La_1.8_NiTiO_6−__δ_.

TEM micrographs (Figure 10) are in good agreement and can be considered as clear evidence of the material degradation after annealing at 1173 K for prolonged periods of time. In the case of the x = 0 composition, 40–80 nm particles are clearly visible coming out of the crystals (Figure 10a), whereas for x = 0.2, large nanoparticles appear combined with some other crystals exhibiting a rather large number of 5–10 nm ex-soluted particles (Figure 10b,c). It must be highlighted that XRD performed in annealed powders reveal changes related with crystallinity loss and the presence of the zirconate phase, though NiO remained mostly unchanged, probably due to a combination of the relatively small amount, particles size and poor crystallinity. This suggest that the primitive perovskite framework remains despite the ex-solution process. However, the exsolution of NiO causes a marked reduction in the crystallinity of the perovskite phase, i.e., the size of the crystalline domains becomes smaller and that combined with misorientation among domains results in broader XRD reflections. This may be a further cause of the response degradation observed in the electrochemical tests. As the powders remain at high temperatures, the structure gradually evolves promoting NiO ex-solution. Under oxidising conditions, NiO exhibits a fairly low conductivity and the gradual concentration increase at the crystal surface does not favour oxygen reduction.

Considering these results, the use of these materials as cathode for SOFC operation at 1173 K is not feasible and should be kept at lower temperatures. Furthermore, this process may occur in other systems where nanoparticle exsolution is considered as a strategy to produce in situ catalysts and boost the electrochemical performance in SOFC electrodes and therefore an evaluation of the materials structure after long-term tests would be desirable.

## 4. Conclusions

The degradation of the polarisation resistance in La_2−x_NiTiO_6−__δ_:YSZ composite electrodes (x = 0, 0.2) for SOFC cathode applications has been assessed by a combination of long-term impedance spectroscopy measurements at 1173 K with XRD and electron microscopy. Although initial tests proved the phase stability and compatibility with 8-YSZ electrolyte below 1473 K, the structural analyses at the end of the long-term test revealed significant changes. Besides clear evidence of amorphization and crystallinity loss, La_2_Zr_2_O_7_ and NiO appeared as secondary phases as further confirmed by XRD, TEM, and SEM. The presence of La_2_Zr_2_O_7_ is compatible with chemical reaction of La_2−x_NiTiO_6−__δ_ and YSZ, whilst NiO is the result of surface ex-solution from the perovskite matrix. The degradation in the polarisation resistance is apparently related with the gradual increase of the cell components resistance. Morphological and geometrical studies of the post-mortem cells revealed that the morphology and porosity of the electrodes are not differentiating factors in the performance of both La_2−x_NiTiO_6−__δ_:YSZ-based symmetrical cells. As the microstructure does not show any signs of delamination, crack formation, particle coarsening or collapse after the electrochemical tests, the gradual increase in the polarisation in La_2−x_NiTiO_6−__δ_ phases is exclusively due to the formation of La_2_Zr_2_O_7_ and NiO that behave as physical barrier. Both phases are poor ionic and electronic conductors, which limit the diffusion processes and the charge transfer processes. These results highlight how relevant degradation tests are when developing alternative electrode materials for SOFCs. The structural evolution or even solid-state reactions may have a dramatic impact in otherwise electrochemical performance in conventional fuel cell tests. This is particularly true in systems such as perovskite showing in situ ex-solution of metal nanoparticles that may evolve towards catalytically inactive phase, such as NiO. The stability of those systems in the long-term should be thoroughly addressed.

## Figures and Tables

**Table 1:** Impedance data of La_2−x_NiTiO_6−__δ_ (x = 0 and 0.2) in the 1173 to 923 K temperature range.

Magnitude	La_2_NiTiO_6_				La_1.8_NiTiO_6−δ_			
	R_O_	R_1_Q_1_	R_2_Q_2_	R_p_	R_O_	R_1_Q_1_	R_2_Q_2_	R_p_
**E_act_ (eV)**	0.85	1.37	1.57	1.52	0.90	0.92	1.66	1.39
**C (F cm^−2^)**	-	8.70 × 10^−5^	1.02 × 10^−4^	-	-	5.67 × 10^−5^	2.82 × 10^−4^	-

**Table 2:** Geometrical analysis of electrode surface from SEM images in LNT and L18NT electrodes; $\bar{x}$, mean value; σ, standard deviation.

Compound	At (μm^2^)	Ap (μm^2^)	ε (%)	As (μm^2^)	Sv (μm^3^)	D	R^2^(D)
La_2_NiTiO_6_							
Image #1	604.002	312.203	52%	1.092	0.858	1.778	0.999
Image #2	546.72	278.213	51%	1.009	0.762	1.772	0.999
Image #3	538.839	288.503	54%	0.895	0.637	1.746	0.999
$\bar{x}$	563.187	292.973	52%	0.999	0.753	1.765	0.999
σ	35.566	17.430	0.01	0.099	0.111	0.017	0.000
La_1.8_NiTiO_6−δ_							
Image #1	601.840	294.525	49%	1.322	1.143	1.796	0.999
Image #2	554.88	287.471	52%	1.086	0.851	1.767	0.999
Image #3	655.191	309.388	47%	1.543	1.442	1.783	1.000
$\bar{x}$	603.970	297.128	49%	1.317	1.146	1.782	0.999
σ	50.189	11.188	0.02	0.229	0.295	0.015	0.000

## Data Availability

The data presented in this study are available on reasonable request from the corresponding author.

## Supplementary Materials

The following are available online at https://www.mdpi.com/article/10.3390/ma14112819/s1, Figure S1: Selected XRD patterns of 1:1 mixtures of La_2−x_NiTiO_6−δ_ (a) x = 0 and (b) 0.2 with YSZ in air at RT (blue), 1073 K (red), 1273 K (pink), and 1473 K (cyan). Bragg peaks (vertical bars) of corresponding phases are found at the bottom (La_2−x_NiTiO_6−δ_ in blue and YSZ in red). Asterisk shows the (222) and (400) diffraction peaks of La_2_Zr_2_O_7_ phase, Figure S2: Relative differences plots (ΔZ_i_) as obtained from the Kramers–Kronig test for La_2_NiTiO_6_ at (a) 1173 K, (b) 1123 K, (c) 1073 K, (d) 1023 K, (e) 973 K, and (f) 923 K, Figure S3: KK transform data in the complex plane (Z′′ vs. Z′) as obtained from the Kramers–Kronig test for La_2_NiTiO_6_ at (a) 1173 K, (b) 1123 K, (c) 1073 K, (d) 1023 K, (e) 973 K, and (f) 923 K, Figure S4: Relative differences plots (ΔZ_i_) as obtained from the Kramers–Kronig test for La_1.8_NiTiO_6__−__δ_ at (a) 1173 K, (b) 1123 K, (c) 1073 K, (d) 1023 K, (e) 973 K, and (f) 923 K, Figure S5: KK transform data in the complex plane (Z′′ vs. Z′) as obtained from the Kramers–Kronig test for La_1.8_NiTiO_6__−__δ_ at (a) 1173 K, (b) 1123 K, (c) 1073 K, (d) 1023 K, (e) 973 K, and (f) 923 K, Figure S6: XRD patterns of La_2−x_NiTiO_6−δ_ (a) x = 0 and (b) 0.2 at 1173 K for 250 h and comparison with corresponding starting materials at room temperature. Asterisk at 2θ ≈ 37.2° indicates the (111) diffraction peak of NiO phase, Figure S7: Comparison of XRD patterns of 1:1 La_2−x_NiTiO_6−δ_:YSZ (a) x = 0 and (b) 0.2 at room temperature (RT, blue) and 1173 K for 15 h (red) and 250 h (green). Asterisks show the (222) and (400) diffraction peaks of La_2_Zr_2_O_7_ phase.
