# Supplementary material for: Analysis of Performance Losses and Degradation Mechanism in Porous La2−X NiTiO6−δ:YSZ Electrodes
Source: Materials (Basel). 2021 May 25;14(11):2819. doi: 10.3390/ma14112819 (PMC8197466; doi:10.3390/ma14112819)
Supplement: Supplementary file 1 [file materials-14-02819-s001.zip › materials-1226231-supplementary.pdf]

materials

MDPI

Article

Analysis of Performance Losses and Degradation Mechanism in Porous La_{2-x} $NiTiO_{6-\delta}$:YSZ Electrodes

Juan Carlos Pérez Flores [1,*], Miguel Castro-García [1], Vidal Crespo-Muñoz [1], José Fernando Valera-Jiménez [1], Flaviano García-Alvarado [2] and Jesús Canales-Vázquez [1,*]

[1] 3D-ENERMAT, Renewable Energy Research Institute, ETSII-AB, University of Castilla-La Mancha, 02071 Albacete, Spain; miguel.castro@uclm.es (M.C.-G.); vidal.crespo@uclm.es (V.C.-M.); josefernando.valera@uclm.es (J.F.V.-J.)

[2] Chemistry and Biochemistry Dpto., Facultad de Farmacia, Universidad San Pablo-CEU, CEU Universities, Boadilla del Monte, 28668 Madrid, Spain; flaga@ceu.es

* Correspondence: JuanCarlos.PFlores@uclm.es (J.C.P.F.); Jesus.Canales@uclm.es (J.C.-V.)

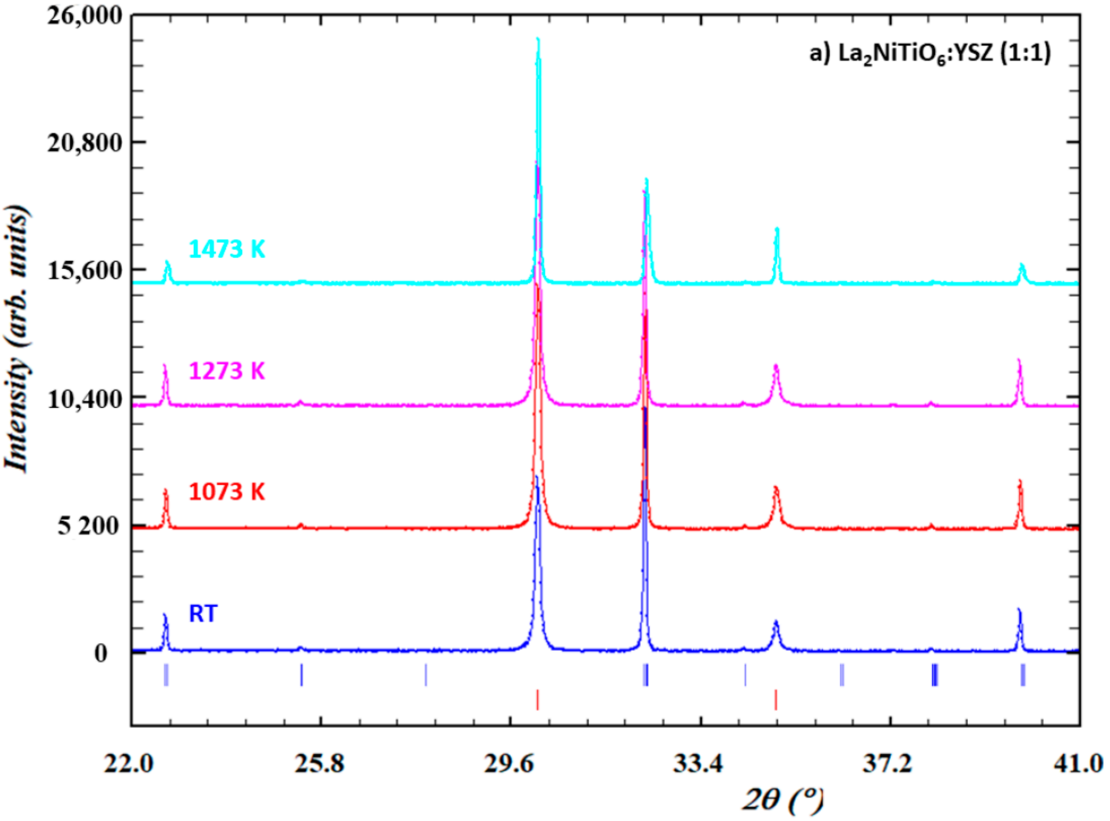

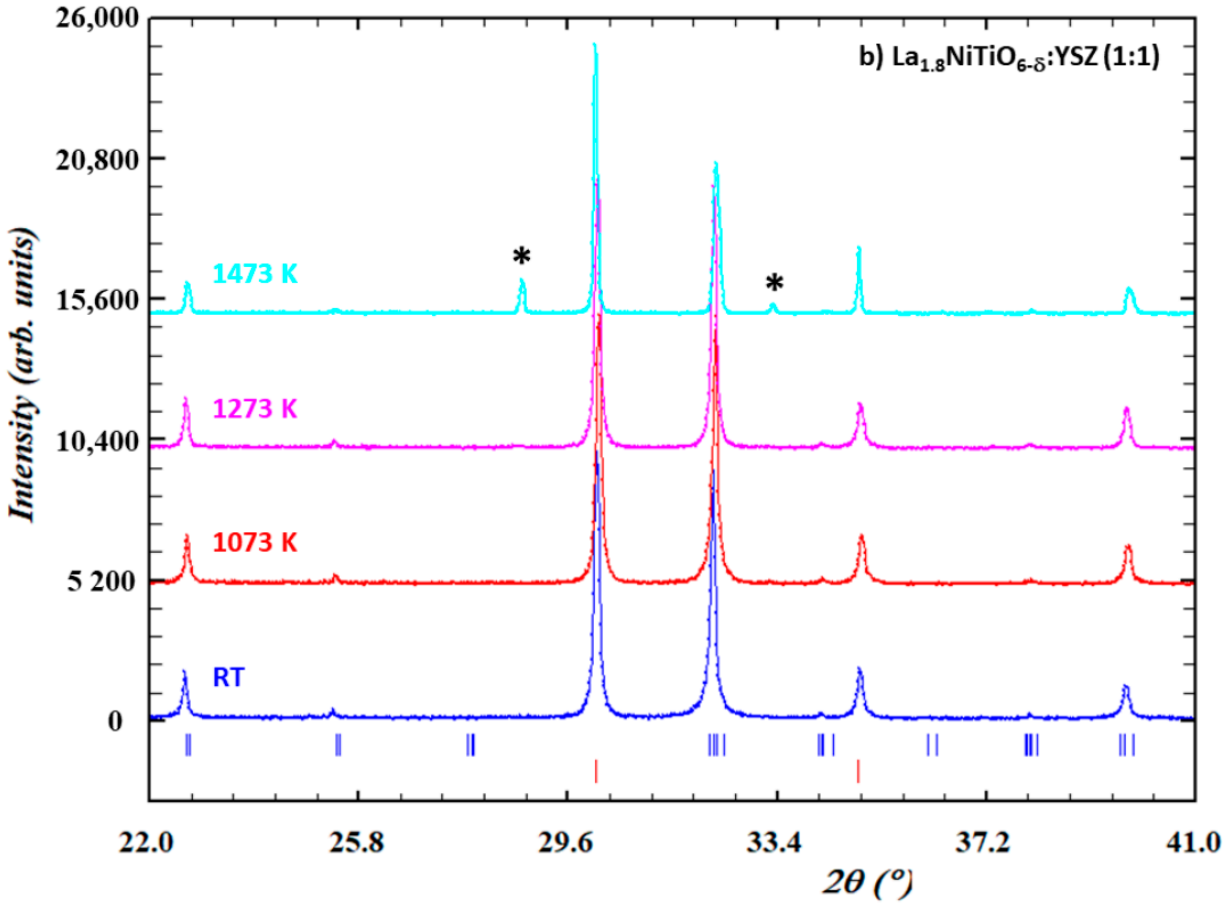

Figure S1. Selected XRD patterns of 1:1 mixture of $La_{2-x}NiTiO_{6-\delta}$ (**a**) x = 0 and (**b**) 0.2 with YSZ in air at RT (blue), 1073 K (red), 1273 K (pink) and 1473 K (cyan). Bragg peaks (vertical bars) of corresponding phases are found at the bottom ($La_{2-x}NiTiO_{6-\delta}$ in blue and YSZ in red). Asterisk shows the (222) and (400) diffraction peaks of $La_2Zr_2O_7$ phase.

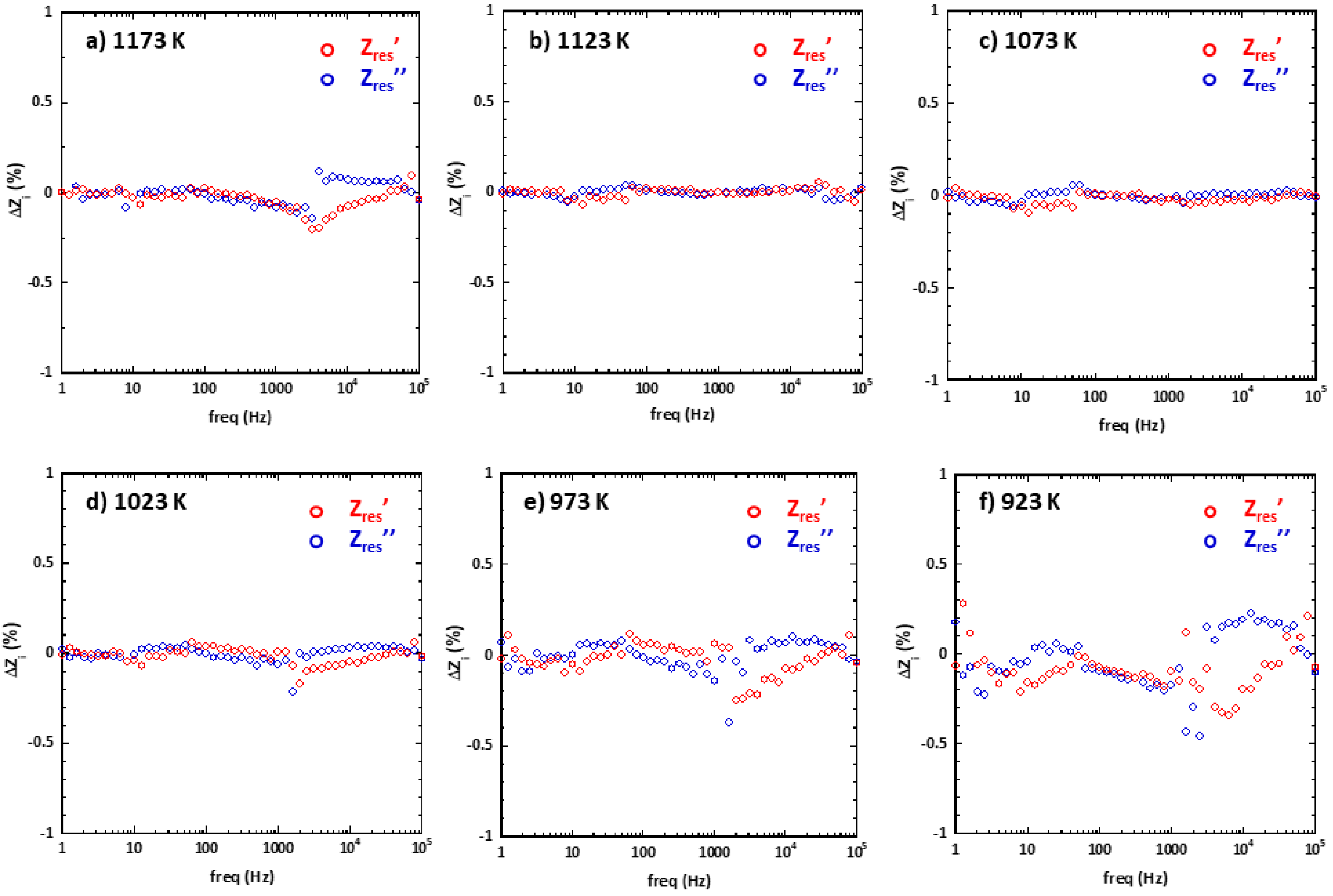

Figure S2. Relative differences plots (ΔZ_i) as obtained from the Kramers-Kronig test for La_2NiTiO_6 at (**a**) 1173 K, **b**) 1123 K, (**c**) 1073 K, (**d**) 1023 K, (**e**) 973 K and (**f**) 923 K.

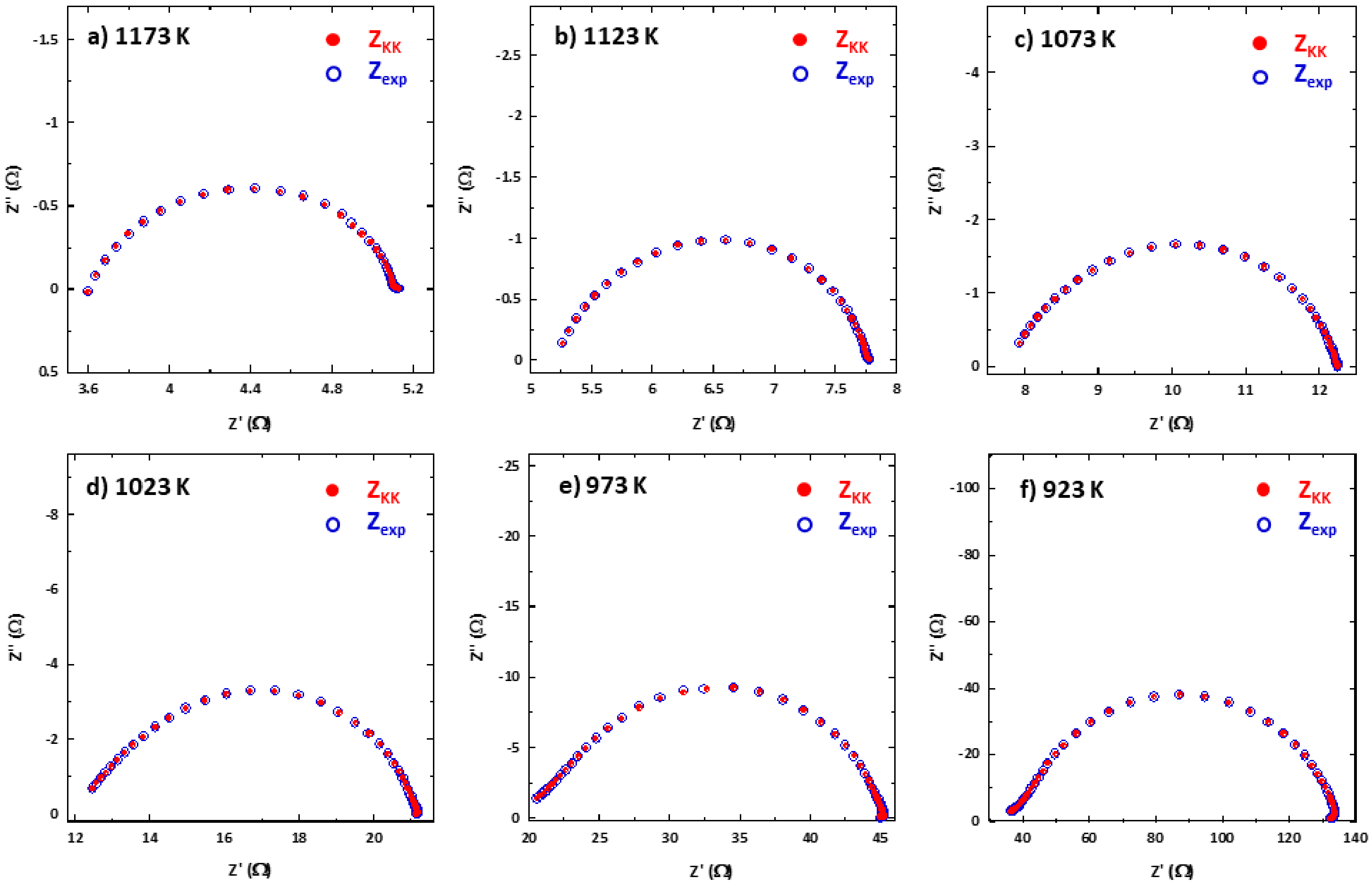

Figure S3. KK transform data in the complex plane (Z′′ *vs.* Z′) as obtained from the Kramers-Kronig test for La_2NiTiO_6 at (**a**) 1173 K, (**b**) 1123 K, (**c**) 1073 K, (**d**) 1023 K, (**e**) 973 K and (**f**) 923 K.

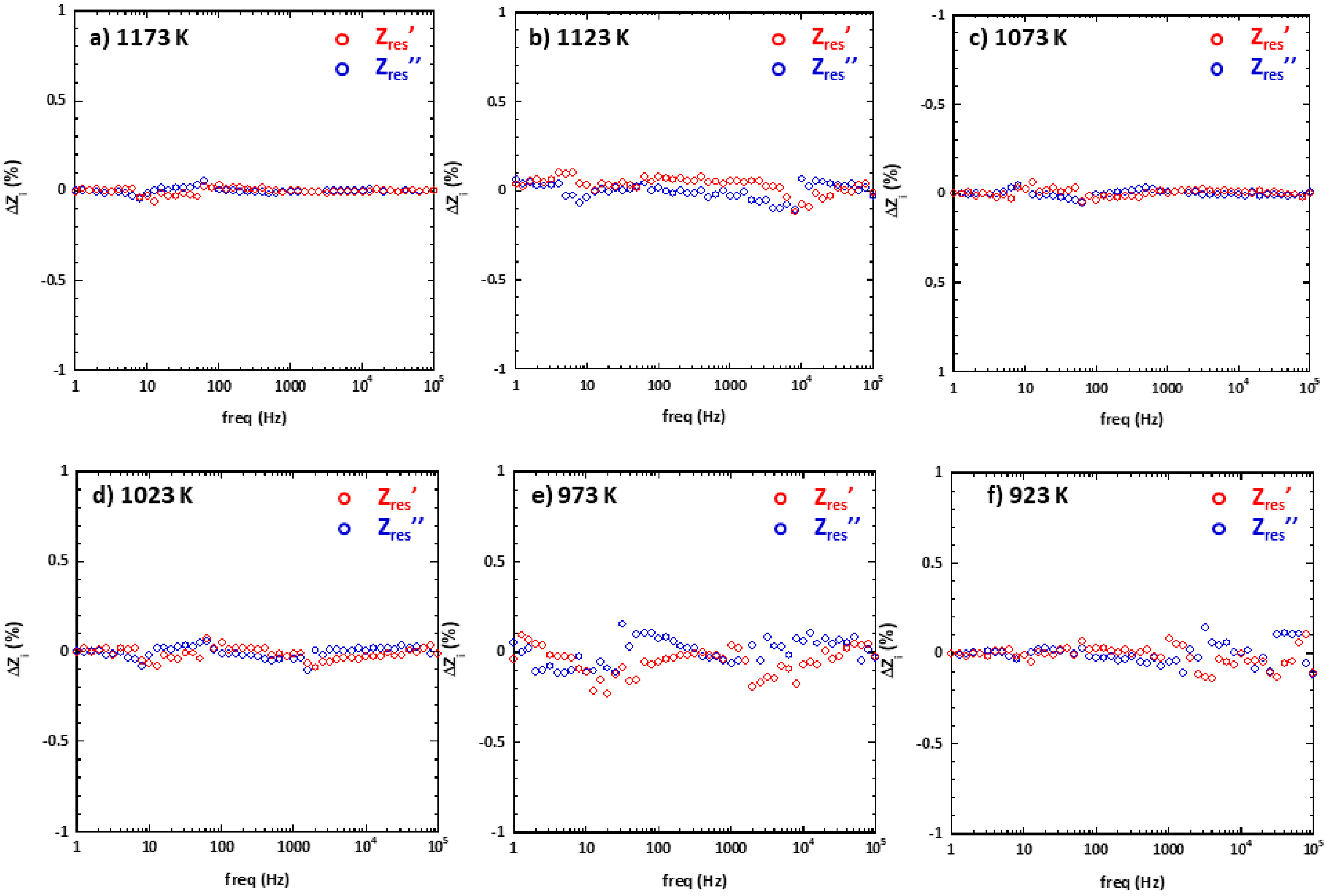

Figure S4. Relative differences plots (ΔZ_i) as obtained from the Kramers-Kronig test for $La_{1.8}Ni$-$TiO_{6-\delta}$ at (**a**) 1173 K, (**b**) 1123 K, (**c**) 1073 K, (**d**) 1023 K, (**e**) 973 K and (**f**) 923 K.

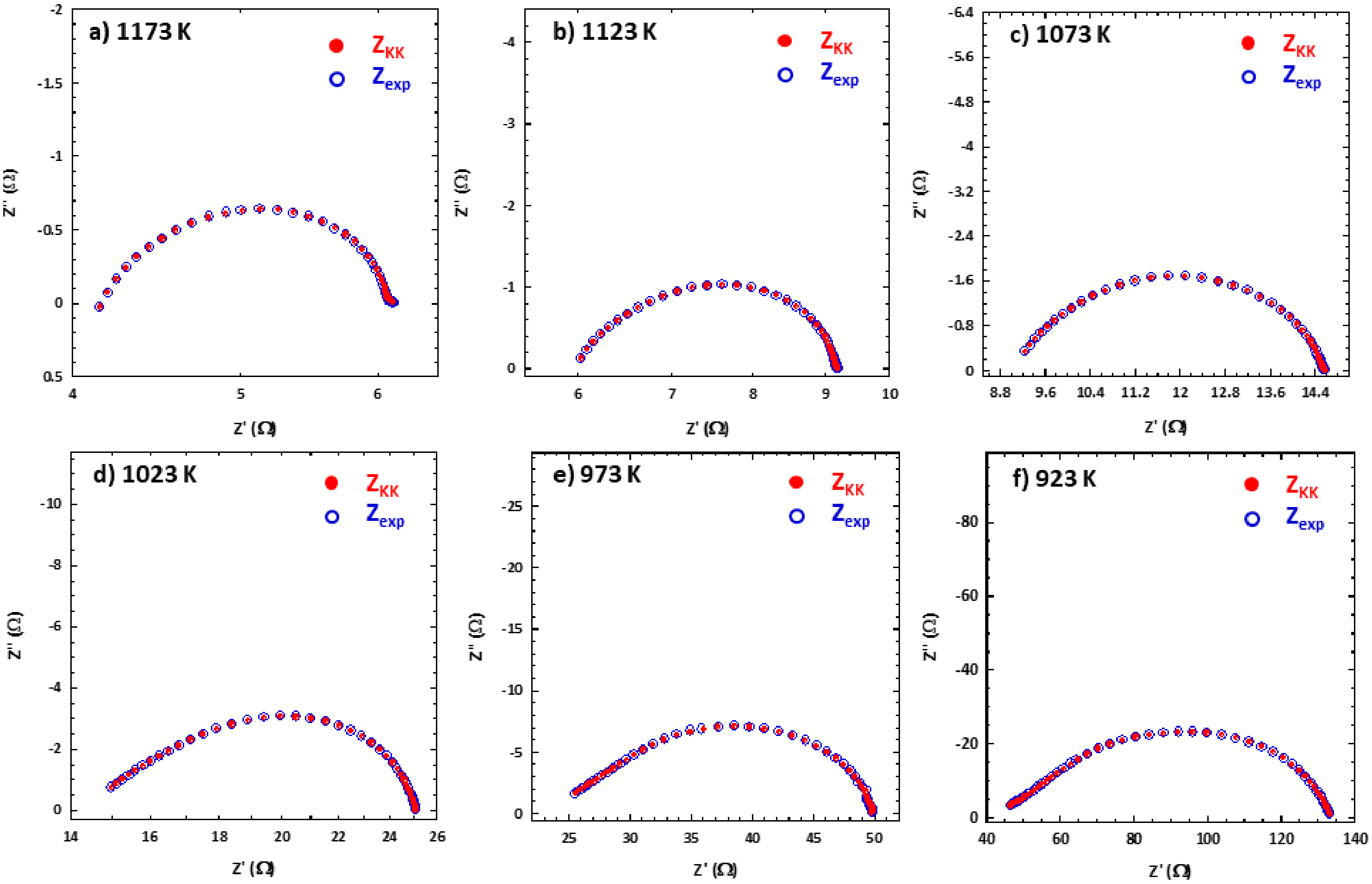

Figure S5. KK transform data in the complex plane (Z′′ *vs.* Z′) as obtained from the Kramers-Kronig test for $La_{1.8}NiTiO_{6-\delta}$ at (**a**) 1173 K, (**b**) 1123 K, (**c**) 1073 K, (**d**) 1023 K, (**e**) 973 K and (**f**) 923 K.

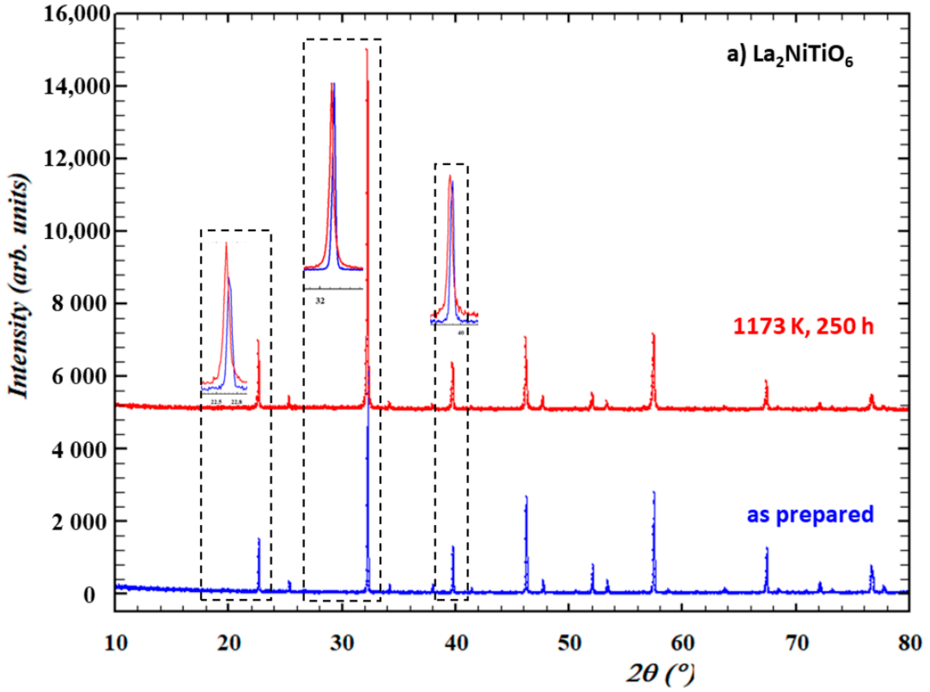

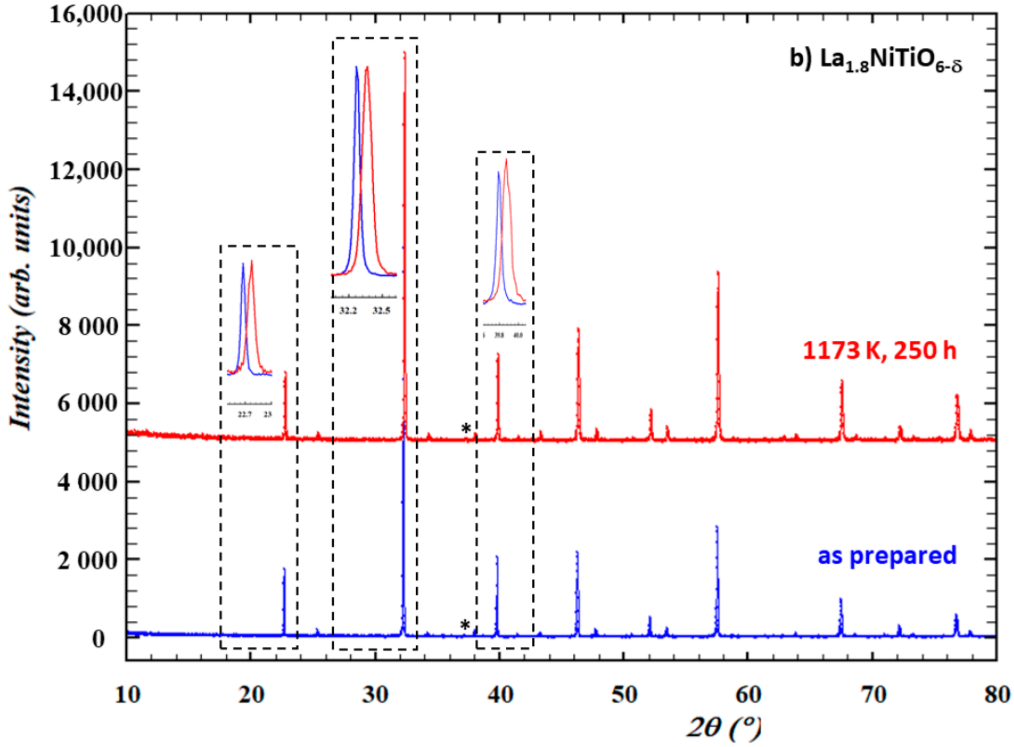

Figure S6. XRD patterns of $La_{2-x}NiTiO_{6-\delta}$ (**a**) x = 0 and (**b**) 0.2 at 1173 K for 250 h and comparison with corresponding starting materials at room temperature. Asterisk at 2θ ≈ 37.2° indicates the (111) diffraction peak of NiO phase.

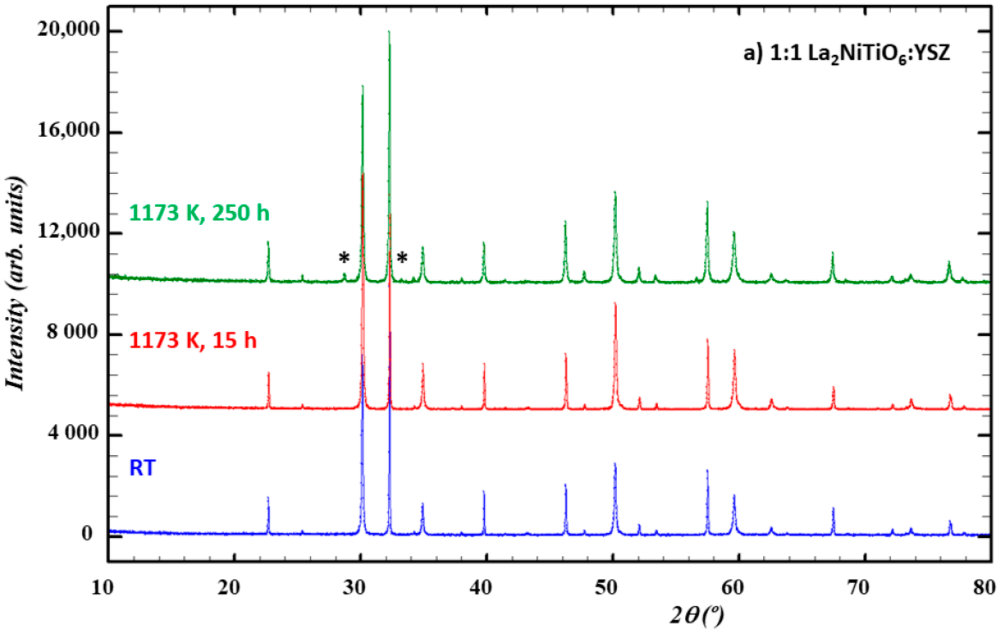

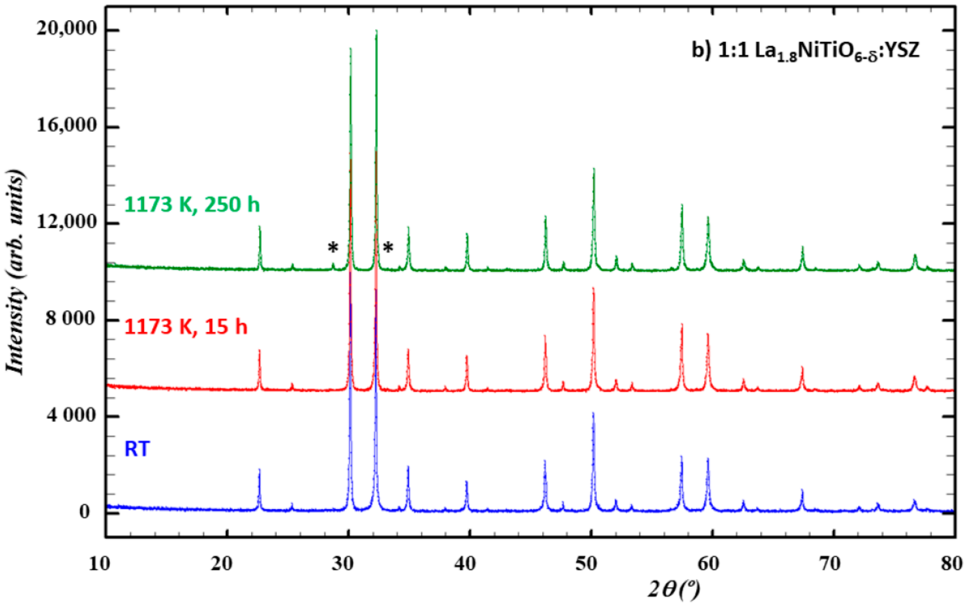

Figure S7. Comparison of XRD patterns of 1:1 $La_{2-x}NiTiO_{6-\delta}$:YSZ (**a**) x = 0 and (**b**) 0.2 at room temperature (RT, blue) and 1173 K for 15 h (red) and 250 h (green). Asterisks show the (222) and (400) diffraction peaks of $La_2Zr_2O_7$ phase.
